# Do plant histone variants stand idly by while DNA viruses invade the nucleus?

**DOI:** 10.1007/s44154-023-00129-7

**Published:** 2023-11-13

**Authors:** Zhihao Jiang, Rosa Lozano-Durán

**Affiliations:** Department of Plant Biochemistry, Center for Molecular Plant Biology (ZMBP), Eberhard-Karls University of Tübingen, Tübingen, Germany

## Main text

In the eukaryotic nucleus, the cellular DNA is always wrapped around protein octamers containing two molecules each of histone H2A, H2B, H3, and H4, forming the nucleosome to compact, protect, organize, and control the genome. Linker histone H1 binds to DNA at the entry and exit sites of the core nucleosome. The N-terminal tails of these histones protrude from the nucleosomes and are the sites of various post-translational modifications, such as methylation, acetylation, phosphorylation, and ubiquitination, all of which jointly modulate gene expression, DNA replication, and DNA repair, among other processes (Foroozani et al. 2022). Interestingly, the organization of DNA into a nucleosomal structure has also been observed in many animal and plant DNA viruses: the genome of nuclear-replicating DNA viruses is also wrapped around nucleosomes using specialized histones encoded by their host or by their own genomes once inside the nucleus to form viral chromatin in what is usually referred to as a minichromosome. The minichromosome is also subject to various epigenetic regulations that control viral replication and gene expression, thereby affecting viral performance (Zarreen and Chakraborty 2020; Balakrishnan and Milavetz 2017).

Histone variants are non-canonical histones that frequently have the capacity to confer specific functional or structural features when incorporated to nucleosomes. Histone variants have been described in all organisms, from yeast to plants and animals, and some variants are conserved throughout eukaryotes, while others are lineage-specific (Foroozani et al. 2022). There are a number of histone variants (such as H2A.Z or H3.3) that can be incorporated into nucleosomes via unique histone chaperones, ultimately modifying their properties (Foroozani et al. 2022). By altering nucleosome composition, histone variants can modify DNA-histone interactions, the internal stability of a nucleosome, as well as the accessibility to chromatin-binding proteins, resulting in a new chromatin landscape, often as a response to developmental cues or diverse environment stresses (Foroozani et al. 2022). Our current understanding of the potential plethora of regulatory roles played by histone variants is, however, limited.

Recent advances in mammalian cells have uncovered the exciting interplay between histone variants and anti-viral immunity, which involves both canonical histones as well as histone variants. The histone cell cycle regulator (HIRA) complex deposits the histone variant H3.3 on invading viral DNA to inhibit viral gene expression and enhance intrinsic anti-viral immunity in human cells (Rai et al. 2017); however, Alvarez-Astudillo et al*.* found that histone H3.3 ectopically expressed in human cells assembles with HIRA into hepatitis B virus (HBV) and enhances viral transcription (Alvarez-Astudillo et al. 2021), suggesting different roles of this variant in virus-host interactions. In HeLa cells, another histone variant, H2A.Z, was found in nucleosomes that occupy the type I interferon (IFN)-stimulated gene promoters, which are removed after IFN stimulation and activate anti-viral responses, suggesting a negative regulatory role for H2A.Z in innate anti-viral immune signaling (Au-Yeung and Horvath 2018). Contrasting with these examples, no reports on the potential interplay between histone variants and infection by plant viruses exist so far.

Geminiviruses, causal agents of devastating diseases in cash and staple crops worldwide, consist of a single-stranded (ss) DNA genome and a limited number of viral proteins (Zarreen and Chakraborty 2020). The geminiviral genome is organized as a minichromosome in the infected plants, and is associated with histones and non-histone proteins. Once the circular ssDNA is released from the capsid, it is converted into a double-stranded DNA (dsDNA) intermediate with the help of host enzymes. This DNA is further processed into covalently closed circular DNA (cccDNA), which is wrapped around nucleosomes to form minichromosomes. Geminiviruses have evolved numerous mechanisms to exploit host epigenetic processes to ensure replication and persistance of the viral genome, while the minichromosome is also a target of host epigenetic regulation to inhibit geminivirus pathogenesis (Zarreen and Chakraborty 2020). Given the diverse roles of histone variants in regulating gene expression also described in plants (Foroozani et al. 2022), as well as multiple lines of evidence implicating histone variants in the regulation of animal virus minichromosome activity, an open question is whether histone variant proteins are deposited on geminivirus minichromosomes and regulate gene expression and viral pathogenesis.

Considering the importance of histone variants in animal-virus interactions, it is tantalizing to speculate that histone variants may indeed also play a role in geminiviral biology. Unique properties distinguish histone variants from canonical histones: for example, histone H3.3 and H2A.Z are found in transcriptionally permissive euchromatic regions, typically overlapping with several active chromatin marks, such as H3K4me3, H3K9me3, or H3K36me3 (Foroozani et al. 2022), some of which have been identified on the geminiviral minichromosome (Zarreen and Chakraborty 2020). The deposition of these histone variants may correlate with the viral requirement for relatively high gene expression to allow for an efficient infection. Moreover, H3.3 allows enrichment of a subset of particularly short genes (< 1 kb) with H3K4me3 (Zhao et al. 2021); viruses tend to contain short genes due to their limited genome size, with geminiviral genes ranging between approx. 84 – 1074 nt. One of the hallmark events of geminivirus infection is the re-entry into the cell cycle to establish a cellular environment permissive for viral DNA replication. H2A.Z.2 drives cell cycle progression by regulating S-phase genes in cancer cells (Vardabasso et al. 2015); if this function is conserved for the homologous histone variant in plants, this could be exploited by geminiviruses, presumably controlling virus fate and multiplication. Intriguingly, transcriptome analyses of *Nicotiana benthamiana* leaves infected with the geminivirus tomato yellow leaf curl virus (TYLCV) show that the expression of genes encoding canonical histones and histone variants is significantly modified (Wu et al. 2019). The histone variants H3.3 and H2A.Z and the canonical histone H1 are strongly up-regulated, whereas most of the other canonical histones are significantly down-regulated, suggesting that geminivirus infection causes dramatic transcriptional changes potentially modifying the cellular pool of these histone proteins (Table 1). Additionally, an apparent lower abundance of histone H2A.1 and H2A, specifically in response to the geminivirus tomato mottle virus, has also been reported in tomato using proteome analysis (Ogden et al. 2020). Along these lines, histone replacement has been suggested to play a potential role in the response of pepper to the geminivirus pepper golden mosaic virus, and more specifically in the recovery process (Gongora-Castillo et al. 2012).

**Table 1:** Differential expression of histone genes in TYLCV local infections in *N. benthamiana*

Gene Name	TYLCV vs EV	GeneID (Niben101Scf-)	Homolog in Arabidopsis	Molecular Function in Arabidopsis
*Histone H3.3a*	Up	02418g00017	AT5G10980	1,2,3
*Histone H2B.5*	Up	04371g02007	AT1G08170	1,2,3
*Histone H2A.Z*	Up	08328g00009	AT1G52740	1,2,3,4
*Histone H1.2*	Down	08111g02008	AT1G14900	5,6,7
*Histone H3.2*	Down	02902g00007	AT1G09200	Unknown
*Histone H2A-IV*	Down	01866g00004	AT5G02560	2,3,5,8
*Histone H3.2*	Down	01150g02009	AT1G09200	Unknown
*Histone H2A.v*	Down	00104g03001	AT3G54560	1,2,3,5
*Histone H3.2*	Down	01150g02010	AT1G09200	Unknown
*Histone H2A.1*	Down	00247g03007	AT1G08880	2,3,5
*Histone H2A.1*	Down	09876g00011	AT5G27670	2,3,5,8
*Histone H3.2*	Down	01094g01011	AT1G09200	Unknown
*Histone H2A.1*	Down	00223g03023	AT5G02560	2,3,5,8
*Histone H2B.1*	Down	00436g10003	AT3G45980	2,3,5
*Histone H2B.3*	Down	05159g00002	AT3G45980	2,3,5
*Histone H2B.1*	Down	07275g00001	AT3G45980	2,3,5
*Histone H2B.1*	Down	05506g00009	AT3G45980	2,3,5
*Histone H3.2*	Down	02216g02012	AT1G09200	Unknown
*Histone H3.2*	Down	05892g00006	AT1G09200	Unknown
*Histone H2A.v*	Down	12159g08001	AT3G54560	1,2,3,5
*Histone H3-like 5*	Down	06080g04011	AT5G65350	2,3
*Histone H3.2*	Down	08833g01006	AT1G09200	Unknown
*Histone H3.2*	Down	09523g01007	AT1G09200	Unknown
*Histone H2A.1*	Down	05676g06016	AT5G02560	2,3,5,8
*Histone H3.1*	Down	06869g02006	AT1G09200	Unknown

RNA-seq data are extracted from (Wu et al. 2019). 1: protein binding; 2: protein heterodimerization activity; 3: structural constituent of chromatin; 4: protein binding; 5: DNA binding; 6: double-stranded DNA binding; 7: nucleosomal DNA binding; 8: chromatin binding. EV: empty vector (used as negative control), Up: up-regulated, Down: down-regulated

On the other hand, the geminiviral infection might also affect the host epigenome, directly or indirectly, through the manipulation of histone variants. Virulence mechanisms evolved to attract or evict histone variants to or from the viral genome might modulate the nuclear availability of these proteins for the plant DNA, and/or interfere with their incorporation, ultimately altering the host transcriptional landscape. The capacity to cause such changes, if they have an effect on viral performance, might also be subjected to selective pressure.

It is by now widely accepted that epigenetics is a crucial battlefield in plant-geminivirus interactions, which compete for their control in the infected cell. However, our understanding of the anti-viral role of this suite of modifications and regulatory processes, as well as their co-option by viruses, is still in its infancy. Considering the known relevance of histone variants in the infection by animal viruses and the hints available in the literature, we believe that exploring the relevance of histone variants for the geminiviral infection is a necessary next step that might not only shed new light on viral pathogenesis and anti-viral defence strategies, but also potentially open new avenues for the generation of future geminivirus-resistant crops.

## Data Availability

Not applicable.
